# British and American Hospitals

**Published:** 1906-08-18

**Authors:** William Epps

**Affiliations:** Secretary, Royal Prince Alfred Hospital, Sydney.


### HOSPITAL ADMINISTRATION.

### CONSTRUCTION AND ECONOMICS.

### BRITISH AND AMERICAN HOSPITALS.

FROM AN AUSTRALIAN STANDPOINT. By William Epps, Secretary, Royal Prince Alfred Hospital, Sydney.

(*Continued from page* 322.)

#### Payment by Patients.

It will have been observed that the so-called "payments" by patients afford a comparatively small proportion of the whole revenue of the hospitals. As a matter of fact, the term "payment" is a misnomer, when considered in relation to the American system. Under the latter, the patients actually do pay, as a rule, sufficient, if not more than enough, to cover the cost of maintenance. In Australia the conditions are different, and we usually discriminate in our financial statements by referring to the amounts received as "contributions." That really states the case. Very rarely does the amount received from the patient equal the cost of his actual maintenance, apart from the fact that he receives medical attendance free. Taken by a large, and as a general principle, we in Sydney put this cost down at about 28s. per week, or 4s. per diem. The amount received from the patient by the hospital is in no sense a fixed one, and depends upon the position of the patient.

Perhaps it may be of some value, by way of example, for me to quote the methods in force at the Royal Prince Alfred, of which I can speak more accurately than of any other hospital. It would be fairly typical of the majority. We do not have the system of hospital letters, which I found in so many English hospitals; but it is the privilege of regular subscribers, medical men, clergymen, justices of the peace, etc., to give to patients letters of recommendation to the effect that to their knowledge the bearers are suitable persons for treatment in a public hospital. The applicant, if well enough, or his representative brings this letter to the Secretary, who questions him as to his circumstances in life—his income, the number of his family, the rent he pays, and so on. These particulars are entered upon his application form, which he then submits, in person, to the medical superintendent or his deputy, who decides as to whether he is a suitable case for admission, medically and from a financial standpoint. If he is approved, the question then arises, What can he pay? He may offer, say, 5s. a week, and the medical superintendent may consider, from the particulars before him, that the payment should be 10s. per week. A little discussion, perhaps, will result in the payment of 7s. 6d., and that is his contribution. Another man may jauntily offer to pay two guineas, and at once raises the suspicion that he is not a suitable case for admission, as being in too good a position for admission to a public hospital. In such case it is more difficult for him to get in than for the man who shows he can only pay 10s. per week. In fact, that is generally the basis of admission. If there be one bed only available, the man who can pay the lowest sum per week, other things being equal, has the preference, as against the man who can pay more. It may be hard in cases, but it is just; though, at the same time, it affects the income of the hospital to a serious extent, and in the future the question may arise whether more income should not be derived from this source.

#### Actual Results.

It may be interesting to readers of The Hospital to know something of the actual results of the system from the hospital standpoint. Approximately, only one-fourth of the patients received are able to, or do, contribute to the cost of their maintenance. All accident or urgent cases are admitted free, and without question, and these represent about one-fifth of the total admissions. The balance, over one-half, are too poor to contribute anything. It should be stated, however, that in the wards no discrimination is made between contributing and free patients. As a matter of fact, the nurses even do not know the financial status of their cases, and there is thus no aristocracy of sickness, as would appear to be the case, to an extent, in American hospitals. For the information of the Board of Directors, I have recently prepared a return showing the actual results of this system on the hospital finances for five years past, which it may be of interest to quote here:—

*Payment from all Sources Compared.*

| Year. | Total patients treated. | Total contributing patients. | Total receipts from patients. | Average payment per week per paying patient. |
|---|---|---|---|---|
| 1901 ... | 3,439 | 911 | £1,838 19 1 | 15s. 7d. |
| 1902 ... | 3,420 | 837 | 2,587 1 8 | 15s. 6d. |
| 1903 ... | 3,461 | 838 | 2,229 0 4 | 13s. 9d. |
| 1904 ... | 3,504 | 865 | 2,433 1 10 | 14s. 3d. |
| 1905 ... | 3,456 | 1,027 | 2,349 11 11 | 11s. 11d. |

It will be seen that there has been a steady decline in the average contributions of patients of late years, which may be due to one or two causes. For example, we had up to twelve months ago been undergoing a period of commercial depression in N.S.W., which doubtless affected the contributory powers of patients; but I fancy a truer reason would be the fact that, in view of the serious demand upon our accommodation—in five years we had a daily average of about 231 patients for 236 beds—there has been a survival of the least fit from a financial standpoint in the competition for admission, and consequently the amounts have decreased.

Last year, as I have stated, we commenced (on June 1) a new system of receiving non-paying patients—other than accident and emergent cases—and instead of a Government payment at per diem have taken all cases coming forward on a bed subsidy system, and many of these patients have been able to contribute something. The general average of amounts received has, however, gone down, though the numbers of contributing patients have increased. As an example of the system it may be of interest if I quote here a weekly return presented to my Board of Directors showing the number and character of patients admitted to the hospital during the previous week. The last return shows the following:—

*Return of Patients in Hospital, March 2, 1906.*

| | | s. | d. | Total. £ | s. | d. |
|---|---|---|---|---|---|---|
| 7 | to pay per week | 2 | 6 | 0 | 17 | 6 |
| 1 | " " | 3 | 0 | 0 | 3 | 0 |
| 3 | " " | 4 | 0 | 0 | 12 | 0 |
| 36 | " " | 5 | 0 | 9 | 0 | 0 |
| 1 | " " | 6 | 0 | 0 | 6 | 0 |
| 4 | " " | 7 | 6 | 1 | 10 | 0 |
| 31 | " " | 10 | 0 | 15 | 10 | 0 |
| 1 | " " | 10 | 6 | 0 | 10 | 6 |
| 1 | " " | 12 | 6 | 0 | 12 | 6 |
| 11 | " " | 15 | 0 | 8 | 5 | 0 |
| 13 | " " | 20 | 0 | 13 | 0 | 0 |
| 4 | " " | 21 | 0 | 4 | 4 | 0 |
| 1 | " " | 30 | 0 | 1 | 10 | 0 |
| 114 | | | | 56 | 0 | 6 |
| 178 | non-paying cases, | | | | | |
| 12 | accident. | | | | | |
| 304 | | | | | | |

The figures given will probably alter steadily in the future. The hospital has now been doubled in size, and at present 318 beds are in occupation and almost all filled; while, before the end of the year, 460 may be available. If an arrangement now under discussion with the Government be carried out, the whole of these beds will be available for the general patients, who now go to the Coast Hospital, but a higher bed subsidy will have to be paid than at present—probably about £40 per bed all round. The reason for this is that, whereas the cost of maintenance will be from £60 to £65 per bed, the difference between this sum and the £35 received from the State would be too great to be made up by income from endowments, subscriptions, and patients' contributions. It has to be remembered that, in this young country, our endowments are not so large as in Great Britain and America, and we have not the large proportion of the wealthy and middle classes to be found there, from whom we may look for annual subscriptions.

### Australian, American, and British Methods.

I fear I have strayed rather from my original intention of giving but a brief outline of the Australian system of contributions by patients, but to make the comparison with British and American methods fully comprehensible, something of the kind appeared to be necessary. Certainly we have evolved a system quite unique, so far as I am aware, in the hospital systems of the world, and one which appears to have been, on the whole, fairly well suited to our conditions. The State has necessarily had to play a great part, as the development of large urban populations in short periods of time, by the aid of immigration, brought about a set of conditions only to be compared to those of Western Canada to-day, and there the State has had to step in and assist. Undoubtedly the tendency is towards a greater reliance upon the State in the matter of hospital finance, and possibly the future may see a change to the system of purely State institutions. I trust this may not be so. State administration becomes too mechanical, and, I venture to say, actually more expensive than the system of independent control, especially where there is a steady fight for existence, as we have all the time, even with large State support.

### Advantages of Voluntary Subscription.

The advantage of the present system is that it maintains alive the spirit of public benevolence, which tends to make the patients in our hospitals more than mere "cases." As matters stand, as much public assistance as possible is secured, and the administration is ultimately in the hands of Boards of Directors, of men of standing in the community, instead of pure officials, and there is thus a safeguard against what might be termed callousness of treatment instead of sympathetic consideration for the position of the sick poor. There is also this great consideration, that the patient who is able to pay even 2s. 6d. a week feels that he is not quite a pauper, and he retains a large proportion of his self respect.

I do not venture to say that the Australian system is better than either the British all-free and all private (as distinct from State or public assistance) or the American scheme of making the wealthier sick pay for their poorer brethren; but it seems to suit our conditions fairly well. So long as we have to fight for our existence, even though we have the State behind us, we have a certain safeguard against extravagance, and at the same time against the sapping of our national self-reliance, and for this reason it is to be hoped the day may be far distant when the State is to be more to us than at present. Should the State system be evolved in its entirety, I fear it would have a tendency to prohibit the employment of the highest medical and surgical skill, as at present, on a gratuitous basis, and tend to the employment of second-class ability of the purely official type.

Perhaps the information I have given may be of some value to those English hospital managers who have not previously had a knowledge of either American or Australian methods, and if so I shall be well repaid for any trouble I have gone to in putting it forward. In a future article I may have an opportunity of referring more in detail to the system of private wards in America and Canada, and to other details of administration in these hospitals, which certainly afford many points of view for careful consideration.

